# The Present Status of the Dental Profession

**Published:** 1870-05

**Authors:** 


### THE PRESENT STATUS OF THE DENTAL PROFESSION.

It is said that labor is honorable. In fact, this has passed into a proverb. But like all proverbs it is susceptible of qualification.

Every kind of labor may be honorable, but the degree of honor, respectability and dignity depends upon how much such labor adds to the refinement and happiness of the human family, and upon the amount of skill, experience, talent, mental culture and learning required in order to successfully perform or practice it.

Hence it is that we use the term "common labor" to express that class of work where little or no mechanical skill and no learning are required; and the term "trade" to designate the class of labor where mechanical dexterity and talent, but no great learning, are necessary to success.

When we approach that class of labor where bone and muscle play only a subordinate part, and where high talents, learning, esthetic conceptions and science and art are called upon to preside over every operation, it is no longer a trade, but comes within the domain of one of the fine arts, or of one of the learned professions.

Dentistry belongs to this class of labor. It is both a science and an art. As a science it is a specialty of medicine as much as surgery. In England this connection is so far recognized that candidates for the Dental degree are required to attend the lectures and be examined by censors appointed by the College of Surgeons. In Prussia, at the University of Berlin, they have a Dental professorship, and a Dental clinic, and students in this department are required to attend the same lectures as the general surgeon. In our own country Dentistry is also recognized as a medical specialty by the better portion of the medical profession.

The Dentist, like the general surgeon, or like the oculist and aurist, must base the superstructure of his practice upon anatomy, histology, physiology, pathology and chemistry. He works upon vitalized structures, endowed with nerves and blood vessels, and through these nerves most intimately connected in sympathy and relationship with every part of the system. With a thorough knowledge of the principles of general medicine, and an innate tact and skill in applying his Dental *materia medica* to a decayed and sick tooth, he arrests the caries—saves the patient, perchance, days of agonizing tooth-ache, and saves the tooth to add to the health, comfort and beauty of its possessor. He performs, in truth, the office of the "Human Healer," and this, says Mr. Carlyle, is "radically a sacred one, and connected with the higher priesthood, or rather being itself the outcome and acme of all priesthoods, and divinest conquest of intellect here below."

The qualifications of the Dentist must embrace even more than this; he must combine with the above requirements the mechanician and the true artist. Cases come up in every day practice where the teeth are not only wanting, but by the absorption of the processes and wasting away of the muscles and tissues of the mouth all natural expression is lost. In restoring these lost parts he is not only compelled to imitate nature, but he has to infuse into his work an expression—*a very life.* He has to depend upon his artistic knowledge and conceptions to enable him to give to the mouth a *speaking* intelligence.

After giving this picture of what Dentistry is, and of what a Dentist should be, let us stop short and ask the question, how does the present status of the Dental profession comport with the requirements of Dentistry, when considered in its scientific and artistic position? Or, in other words, has the mass of the Dental mind, during the last ten years, been tending upward toward a standard of professional dignity and preferment, or has it been downward toward a simple mechanical routine or common trade? These questions each one can answer for himself, after a few of the obstacles to true Dental progress have been considered.

One great obstacle to Dental progress has been the introduction of cheap and common materials as a tooth-base to the exclusion of those metals and modes of construction that demand talent and skill. In the practice of hundreds of Dentists, rubber, for instance, is the only thing used.

I wish it to be understood, that I do not condemn the use of rubber *in toto.* It will always maintain an important position in Dental prosthesis, for example, in the construction of artificial palates, obturators, splints for broken jaws, etc. But I *do* condemn and abominate the abuse of it.

The advent of rubber work in Dentistry has been the means of not only lowering the grade of qualification and giving encouragement to men to enter the profession without any preparatory training and education, but it has also caused the people to pay less attention to the preservation of the natural teeth. As it requires but little brain, skill and ability to work, these Dental novitiates (not to use any stronger yet more appropriate term) have laid hold of it and recommend it to their victims as better and more durable than gold and all other kinds of work. To be able to take an impression—make a model—stick upon it with wax some block or section teeth which automatically arrange themselves—make a mould and stuff it full of prepared caoutchouc—steam it until the rubber hardens—scrape off the plaster and then insert the piece into the mouth, is the extent of the Dental attainments of many of these rubber specialists. With them this is Dentistry. With no higher conceptions of the profession which they disgrace, they pull out indiscriminately all the natural teeth they can and insert rough, clumsy, worthless abortions of rubber, or something else of like character, which libels the countenance of the unfortunate subject. To call Dentistry a profession under such circumstances is preposterous. It is making it a very common trade.

Many people know very little what Dentistry is, and they argue thus: if they can get a set of false teeth, *warranted not to ache*, for a deal less money than they would have to pay a good operator to save their natural ones, they will let these be destroyed by caries. This has, beyond doubt, been one of the causes of the fearful increase, during the last few years, of the ravages of Dental decay in this country.

To remove this standing disgrace to Dental science some practitioners recommend the abandonment of mechanical Dentistry entirely to these quacks, supposing thereby that the people, after a time, will look in disgust upon artificial teeth, and will pay so much attention to the preservation of the natural teeth that there will be no need of artificial substitutes. This is a very pleasing thought, but, alas, its *actuality* is so very far off in the distant future, that I fear unless human nature is radically changed, both physically and mentally, it will never be realized. The Dental profession can not afford to stake its reputation and advancement upon any such chimerical theories. In spite of all the physiological and hygienic preaching, artificial teeth will be required. Hence, why shun the mechanical department of our profession and allow it to be a common vehicle for pretenders to enter our ranks? The sensible course for every true Dentist to pursue is to go to work and raise the status of mechanical Dentistry by introducing and recommending those materials for artificial work upon which the highest talent can be displayed. We must raise the standard of our work so high that bunglers and quacks can not reach it.

Dental progress has also been retarded, and professional respectability lowered by a dereliction of duty and want of faithful watching on the part of the sentinels who guard the entrance to our profession. Men are admitted to share its honors who have neither the natural talents nor the requisite education for the business. This is partly owing to a disregard of the moral duty and responsibility resting on those Dentists who receive students for private instruction. Many persons look upon Dentistry as a thing of easy acquisition, and all that is requisite in order to gain a knowledge of it is to go into some Dental office and laboratory, and witness a few manipulations, procure a few instruments, and then they are ready to assume the duties of a full practice. Now, there are too many Dentists who, instead of disabusing such persons of their wrong ideas, encourage them, and offer to teach them for a few paltry dollars everything pertaining to the business in a few weeks or months.

You may teach a pupil all the rules of drawing and perspective—the laws of coloring, and furnish him with all the necessary paints, brushes and materials to produce a picture, but unless he possesses the proper mental organization he never can be made an artist. Precisely so it is with the Dentist. If he has not the necessary talents, all the private instruction, colleges and practice will not make him a good operator. Hence it behooves every Dentist who has the good of his profession at heart, not to receive any pupil, nor to encourage and persuade any person to become such, who has not the proper basis (including good moral character) upon which to build a course of professional training. No pupil should be taken for a less term than three years, including medical or Dental lectures. During the first year, a systematic course of reading and examinations should be pursued, including a training in some of the minor operations of the laboratory. The second and third years should be devoted to reading, examinations, lectures, manipulations in the laboratory, with as much operating as circumstances will allow.

Before closing this paper duty compels me to speak of one other evil which is doing immense mischief in lowering the status of our profession in this country in the eyes of the medical profession, and also in the esteem of intelligent Dentists abroad. It is the *cheapening* (if I may so speak) of the grade of instruction imparted at our Dental Colleges by a spirit of rivalry. This has become so notorious, that a Dental diploma, as an index of qualification, is, now-a-days, hardly worth the parchment upon which it is printed. The mania that has lately sprung up in the profession of starting new Dental colleges can only be attended with injurious consequences. Some of the bad effects we have all seen by the *expose* made of some of these short-lived institutions in the way of bestowing honors. The profession does not need *more* colleges—it *needs less.* One North, one South, and one West would be amply sufficient. The faculties of these could then be composed of the best talent, and pecuniarily sustained. Able professors could then devote their whole time and attention to the duties devolving on them. *

